# Neuroprotective effect of selumetinib on acrolein-induced neurotoxicity

**DOI:** 10.1038/s41598-021-91507-6

**Published:** 2021-06-14

**Authors:** Hui-Ju Huang, Hsiang-Tsui Wang, Ting-Yu Yeh, Bo-Wei Lin, Young-Ji Shiao, Yu-Li Lo, Anya Maan-Yuh Lin

**Affiliations:** 1 Department of Medical Research, Taipei Veterans General Hospital, Taipei, Taiwan; 2 Institute of Pharmacology, National Yang Ming Chiao Tung University, Taipei, Taiwan; 3 National Research Institute of Chinese Medicine, Ministry of Health and Welfare, Taipei, Taiwan; 4 Department of Pharmacy, National Yang Ming Chiao Tung University, Taipei, Taiwan

**Keywords:** Drug discovery, Neuroscience, Diseases

## Abstract

Abnormal accumulation of acrolein, an α, β unsaturated aldehyde has been reported as one pathological cause of the CNS neurodegenerative diseases. In the present study, the neuroprotective effect of selumetinib (a MEK–ERK inhibitor) on acrolein-induced neurotoxicity was investigated in vitro using primary cultured cortical neurons. Incubation of acrolein consistently increased phosphorylated ERK levels. Co-treatment of selumetinib blocked acrolein-induced ERK phosphorylation. Furthermore, selumetinib reduced acrolein-induced increases in heme oxygenase-1 (a redox-regulated chaperone protein) and its transcriptional factor, Nrf-2 as well as FDP-lysine (acrolein-lysine adducts) and α-synuclein aggregation (a pathological biomarker of neurodegeneration). Morphologically, selumetinib attenuated acrolein-induced damage in neurite outgrowth, including neuritic beading and neurite discontinuation. Moreover, selumetinib prevented acrolein-induced programmed cell death via decreasing active caspase 3 (a hallmark of apoptosis) as well as RIP (receptor-interacting protein) 1 and RIP3 (biomarkers for necroptosis). In conclusion, our study showed that selumetinib inhibited acrolein-activated Nrf-2-HO-1 pathway, acrolein-induced protein conjugation and aggregation as well as damage in neurite outgrowth and cell death, suggesting that selumetinib, a MEK–ERK inhibitor, may be a potential neuroprotective agent against acrolein-induced neurotoxicity in the CNS neurodegenerative diseases.

## Introduction

Due to the abundance of polyunsaturated fatty acids which are the targets of lipid peroxidation, the brain is reportedly vulnerable to lipid peroxidation^1,2^. Acrolein, an α, β-unsaturated aldehyde, is one of the metabolites of lipid peroxidation^3,4^. Due to its highly reactive activity, acrolein is capable of damaging HT22 hippocampal cells^5^ and human neuroblastoma SH-SY5Y^6^ as well as primary cultured cells from dorsal root ganglion and sympathetic ganglion^7^. Furthermore, acrolein is pro-neuroinflammatory when treating C6 astrocytes, BV-2 cells^8^ and primary cultured microglia^9,10^. Animal studies further support a neurotoxic role of acrolein in experimental animals subjected to spinal cord injury (SCI), stroke and intranigral infusion of acrolein^11–13^. Accordingly, high levels of acrolein and acrolein-protein adducts contribute to the pathophysiology of CNS neurodegenerative diseases^14–17^.

Mounting evidence has supported several neurotoxic mechanisms underlying acrolein-induced injury. Due to its electrophilic activity, acrolein reportedly attacks DNA to form acrolein/guanosine adducts which enhance the cross-linking of DNA with itself or with proteins^14^. At the same time, acrolein is capable of affecting proteins pathologically by conjugating proteins as FDP-lysine adducts, enhancing hyperphosphorylation of tau proteins and forming α-synuclein aggregates^3,11,18^. Furthermore, acrolein induces oxidative responses, including lipid peroxidation, free radical generation, and mitochondrial damages^6,9,11^. In addition, acrolein is pro-inflammatory by activating a disintegrin and metalloproteinase domain-containing protein-10, inducing inflammasome formation and releasing pro-inflammatory cytokines in activated astrocytes and microglia^8,9,19^. Both in vitro and in vivo studies consistently demonstrated acrolein-induced neurotoxicity of cultured nerve cells and neurodegeneration of experimental animals which mimicked disease progression. Non-clinical studies have employed acrolein scavengers, including dimercaprol and hydralazine to ameliorate acrolein-induced neurotoxicity in the experimental models of SCI and Alzheimer’s disease^12,20–22^. However, no clinical application of neuroprotective agents, including acrolein scavengers, against acrolein-related neurotoxicity has been issued.

To search novel neuroprotective strategies for acrolein-induced neurotoxicity, it is crucial to first delineate a druggable target, such as acrolein-activated signaling transduction, including PI3K-AKT and MAPK pathways. Previous studies have shown that acrolein increased^6^ or decreased phosphorylated AKT levels in vitro^23^. In contrast, acrolein consistently elevated phosphorylated ERK levels in human neuroblastoma SH-SY5Y cells, HT22 mouse hippocampal cells and BV-2 cells^5,6,9,19^. Several studies have employed MEK–ERK inhibitors, including PD98059 and U0126, to abolish acrolein-induced ERK phosphorylation and cell death^5,6,19^, indicating the therapeutic potentials of ERK inhibitors on acrolein-induced neurotoxicity^5^. To support this notion, our previous study showed that selumetinib (AZD 6244), an anti-cancer drug with a MEK–ERK inhibitory activity attenuated acrolein-induced neuroinflammation in BV-2 cells^9^. Furthermore, selumetinib has been shown to reduce neuropathic pain in rats subjected to chronic constriction injury^24^ and may be beneficial to frontotemporal dementia in vitro^2^. In the present study, the aim was two-fold. One was to delineate the involvement of MEK–ERK signaling in the acrolein-induced neurotoxicity using primary cultured cortical neurons. The other was to study the neuroprotective effect of selumetinib on acrolein-induced neuronal toxicity.

## Results

### MEK–ERK activation in the acrolein-induced neurotoxicity

**Figure 1:**
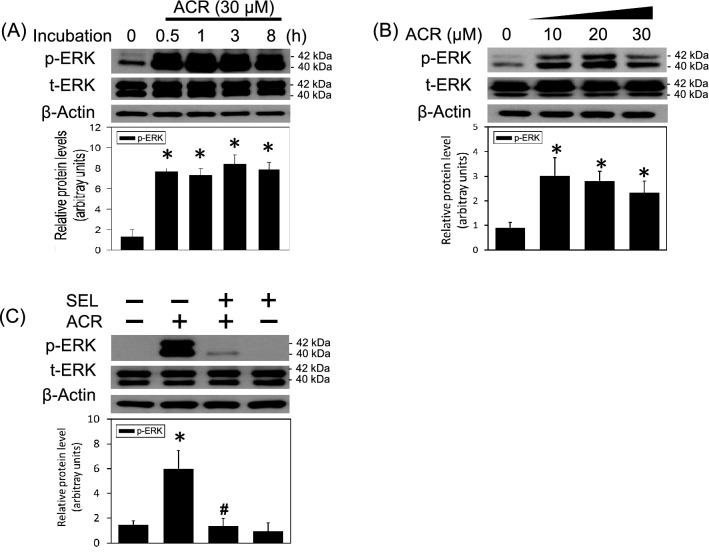
Effect of selumetinib on acrolein-elevated ERK phosphorylation. (**A**) Primary cultured cortical neurons were treated with acrolein (30 μM) for 0.5–8 h. (**B**) Primary cultured cortical neurons were treated with acrolein (0–30 μM) for 24 h. (**C**) Primary cultured cortical neurons were treated with acrolein (30 μM) with/without selumetinib (SEL, 10 μM) for 24 h. Western blot assay was employed to measure total ERK (t-ERK) and phosphorylated ERK (p-ERK). Each lane contained 30 μg protein for all experiments. Graphs show statistical results from relative optical density of bands on the blots. Values are the mean ± SEM. (n = 3/treatment). *p < 0.05 statistically significant in the acrolein groups compared with the control groups; ^#^p < 0.05 statistically significant in acrolein plus SEL group compared with acrolein group by one-way analysis of variance (one-way ANOVA) and followed by the LSD test as post-hoc method. The original Western Blot films of Figures 1 to 6 can be found in the Supplementary Information.

In the present study, the involvement of MEK–ERK signalings in the acrolein-induced neuronal toxicity was delineated using primary cultured cortical neurons. A time-dependent effect of acrolein (30 μM) on ERK phosphorylation was studied. We found that incubation of acrolein for 30 min significantly increased phosphorylated ERK levels (40 and 42 kDa) and maintained the elevated ERK phosphorylation for 8 h (Fig. 1A) and 24 h (Fig. 1B). Furthermore, acrolein concentration-dependently increased ERK phosphorylation in primary cultured cortical neurons (Fig. 1B). The involvement of a MEK–ERK signaling in acrolein-induced neuronal toxicity was delineated using selumetinib, a non-ATP competitive MEK inhibitor. We found that co-incubation with selumetinib (10 μM) significantly attenuated acrolein-induced ERK phosphorylation (Fig. 1C), indicating that the MEK–ERK signaling pathway appears to be involved in acrolein-induced neuronal toxicity.

### Selumetinib inhibited acrolein-induced oxidative stress, protein conjugation and aggregation

**Figure 2:**
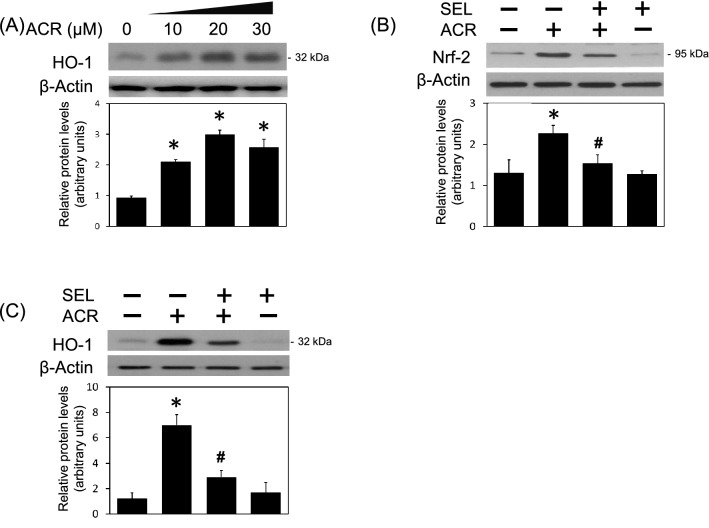
Effect of selumetinib on acrolein-induced oxidative stress. (**A**) Primary cultured cortical neurons were treated with acrolein (0–30 μM). (**B**,**C**) Primary cultured cortical neurons were treated with acrolein (30 μM) with/without selumetinib (SEL, 10 μM) for 24 h. Western blot assay was employed to measure HO-1 (**A**), Nrf-2 (**B**) and HO-1 (**C**). Each lane contained 30 μg protein for all experiments. Graphs show statistical results from relative optical density of bands on the blots. Values are the mean ± SEM (n = 3/treatment). *p < 0.05 statistically significant in the acrolein groups compared with the control groups; ^#^p < 0.05 statistically significant in acrolein plus SEL group compared with acrolein group by one-way analysis of variance (one-way ANOVA) and followed by the LSD test as post-hoc method.

**Figure 3:**
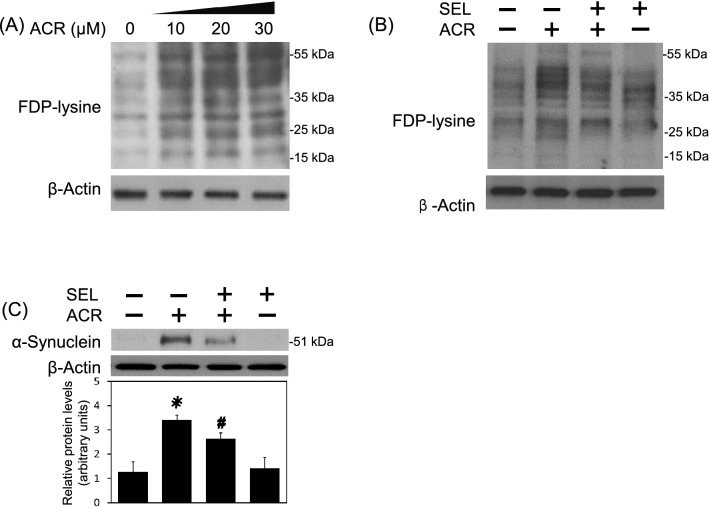
Effect of selumetinib on acrolein-induced protein conjugation and α-synuclein aggregation. (**A**) Primary cultured cortical neurons were treated with acrolein (0–30 μM) for 24 h. Western blot assay was employed to measure FDP-lysine. Each lane contained 25 μg protein for all experiments. Representative Western blot shows the effect of acrolein on FDP-lysine. Similar experiments were performed in duplicate. (**B**,**C**) Primary cultured cortical neurons were treated with acrolein (30 μM) with/without selumetinib (SEL, 10 μM) for 24 h. Western blot assay was employed to measure FDP-lysine (**B**) and α-synuclein (**C**), respectively. Each lane contained 30 μg protein for all experiments. Graphs show statistical results from relative optical density of bands on the blots. Values are the mean ± SEM. (n = 3/treatment). *p < 0.05 statistically significant in the acrolein groups compared with the control groups; ^#^p < 0.05 statistically significant in acrolein plus SEL group compared with acrolein group by one-way analysis of variance (one-way ANOVA) and followed by the LSD test as post-hoc method.

Oxidative stress is known as one major mechanism underlying acrolein-induced neurotoxicity. Indeed, incubation of acrolein increased HO-1 levels (a redox-regulated enzyme) in a concentration-dependent manner in the primary cultured cortical neurons (Fig. 2A). Co-incubation of selumetinib attenuated acrolein-induced elevation in Nrf-2 (Fig. 2B, a transcriptional factor of HO-1 expression) and HO-1 (Fig. 2C), suggesting that MEK–ERK signaling pathway is responsible for acrolein-induced oxidative stress. Furthermore, acrolein concentration-dependently increased FDP-lysine (acrolein-lysine adducts) in primary cultured cortical neurons (Fig. 3A). Co-incubation of selumetinib attenuated acrolein-induced increases in FDP-lysine levels in primary cultured cortical neurons (Fig. 3B). At the same time, selumetinib attenuated acrolein-induced formation of α-synuclein trimer at 51 kDa (Fig. 3C, a pathological biomarker of neurodegeneration). These data indicate that the MEK–ERK pathway is responsible for acrolein-induced protein conjugation and aggregation.

### Selumetinib attenuated acrolein-induced loss of neurite outgrowth

**Figure 4:**
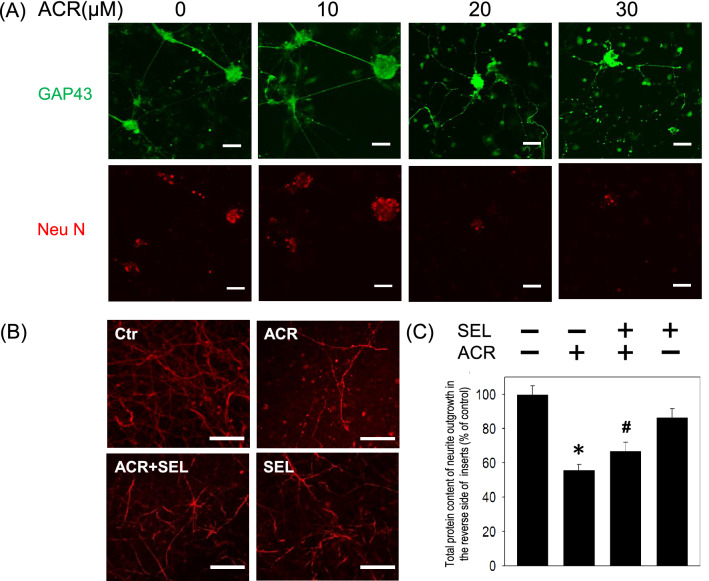
Effect of selumetinib on acrolein-induced reduction in neurite outgrowth. (**A**) Primary cultured cortical neurons (3 × 10^5^ cells in a 35 mm petri dish containing coverslips) were treated with acrolein (0–30 μM) for 24 h. Representative immunofluorescent data using GAP-43 antibody show the effect of acrolein on neurite outgrowth. Caliber: 50 μm. (**B**) Primary cultured cortical neurons were cultured in Transwell inserts and treated with acrolein (30 μM) with/without selumetinib (SEL, 10 μM) for 24 h. Representative immunofluorescent data using MAP-2 antibody show the neurites on the reverse side of the Transwell insert. Caliber: 50 μm. (**C**) Statistical data showed the effect of selumetinib on acrolein-induced reduction in total protein content of neurite outgrowth grown on the other side of Transwell inserts. Values are the mean ± SEM. (n = 3/treatment). *p < 0.05 statistically significant in the acrolein groups compared with the control groups; ^#^p < 0.05 statistically significant in acrolein plus SEL group compared with acrolein group by one-way analysis of variance (one-way ANOVA) and followed by the LSD test as post-hoc method.

To investigate the effect of acrolein on the neurite outgrowth, the morphology of the primary cultured cortical neurons was studied. Compared with the vehicle-treated cells, our immunostaining data using GAP-43 antibody showed that acrolein concentration-dependently damaged neurite outgrowth (Fig. 4A). A low concentration of acrolein (10 μM) for 8 h did not cause significant changes in the neurite outgrowth. However, higher concentrations of acrolein (20–30 μM) caused focal bead-like swellings, neuritic beading and discontinuation of neurites (Fig. 4A). Furthermore, a trans-well insert was employed to culture primary cultured cortical neurons and allowed neurites extending to the reverse side of the trans-well insert. The immunostaining study using MAP-2 antibody showed that co-incubation with selumetinib prevented acrolein-induced reduction in neurite outgrowth on the reverse side of the trans-well insert (Fig. 4B). At the same time, selumetinib attenuated acrolein-induced reduction in total protein content of the neurites on the reverse side of the trans-well insert (Fig. 4C). Our data indicate that the MEK–ERK signaling pathway is responsible for acrolein-induced damage of neurite outgrowth of primary cultured cortical neurons.

### Selumetinib prevented acrolein-induced programmed cell death

**Figure 5:**
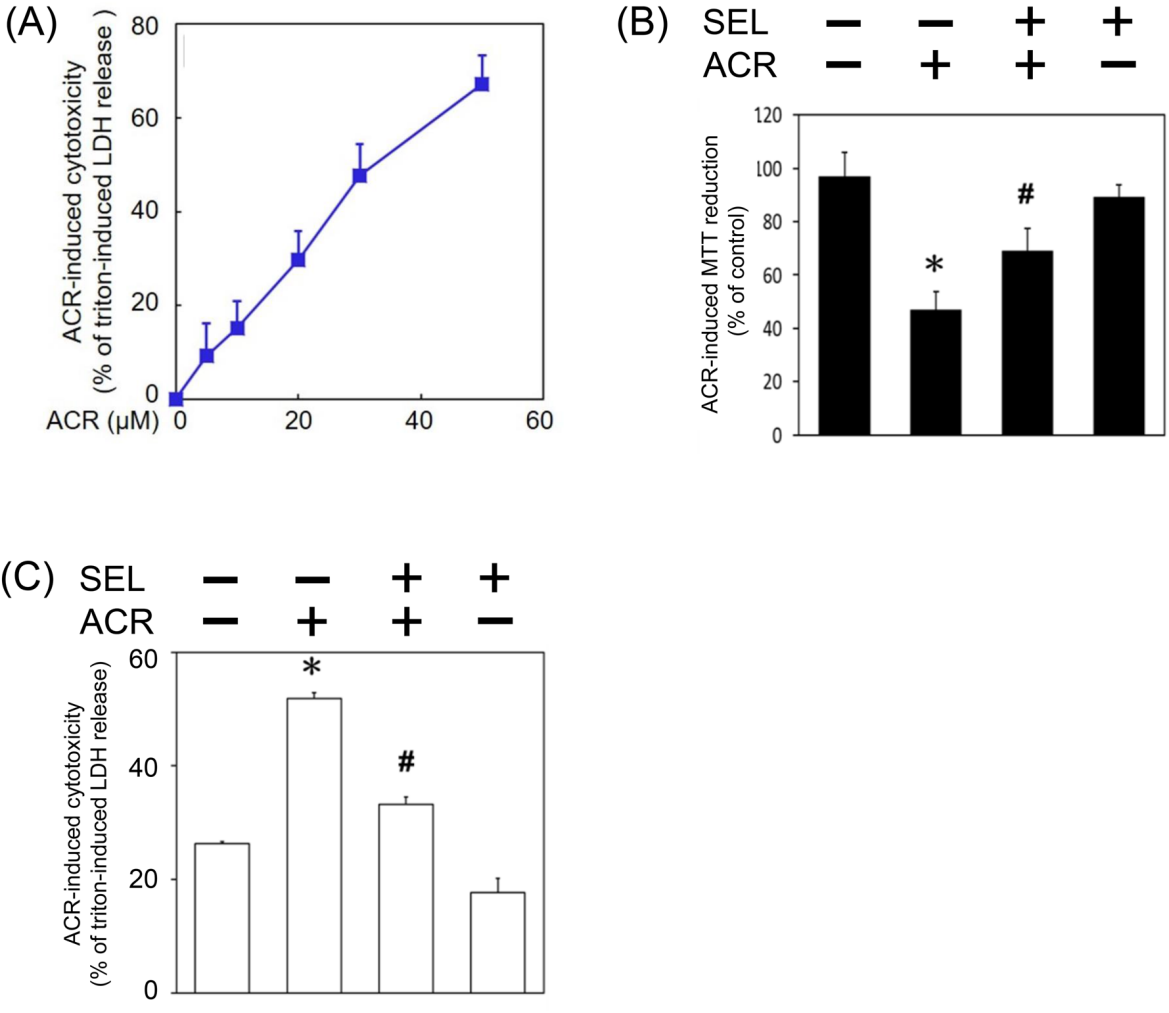
Effect of selumetinib on acrolein-induced cytotoxicity. (**A**) Primary cultured cortical neurons (5 × 10^6^ cells /well) in a 24-well plate were treated with acrolein (0–50 μM) for 24 h. Cell death was measured with LDH assay. Values are the mean ± SEM (n = 3/group). *p < 0.05, statistically significant in the acrolein groups compared with the control groups by *t* test. (**B**,**C**) Primary cultured cortical neurons were treated with acrolein (30 μM) with/without selumetinib (SEL, 10 μM) for 24 h. Cell viability was measured using MTT assay (**B**) and cell death using LDH assay (**C**), respectively. Values are the mean ± SEM (n = 3/group). *p < 0.05 statistically significant in the acrolein groups compared with the control groups; ^#^p < 0.05 statistically significant in acrolein plus SEL group compared with acrolein group by one-way analysis of variance (one-way ANOVA) and followed by the LSD test as post-hoc method.

**Figure 6:**
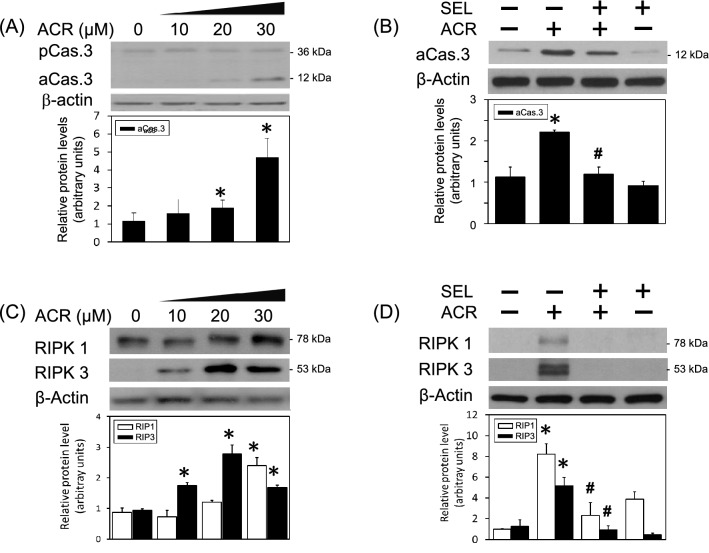
Effect of selumetinib on acrolein-induced programmed cell death. (**A**) Primary cultured cortical neurons were treated with acrolein (ACR, 0–30 μM) for 24 h. (**B**) Primary cultured cortical neurons were treated with acrolein (30 μM) with/without selumetinib (SEL, 10 μM) for 24 h. (**C**) Primary cultured cortical neurons were treated with acrolein (ACR, 0–30 μM) for 24 h. (**D**) Primary cultured cortical neurons were treated with acrolein (30 μM) with/without SEL (10 μM) for 24 h. Western blot assay was employed to measure active caspase 3 (aCas.3), RIP1 and RIP3. Each lane contained 30 μg protein for all experiments. Values are the mean ± SEM (n = 3/treatment). *p < 0.05 statistically significant in the acrolein groups compared with the control groups; ^#^p < 0.05 statistically significant in acrolein plus SEL group compared with acrolein group by one-way analysis of variance (one-way ANOVA) and followed by the LSD test as post-hoc method.

In addition, the cytotoxic effect of acrolein was investigated by measuring cell death using LDH assay and cell viability using MTT assay, respectively. We found that acrolein concentration-dependently induced cell death of primary cultured cortical neurons (Fig. 5A). Moreover, selumetinib diminished acrolein-reduced cell viability and acrolein-induced cell death of primary cultured cortical neurons (Fig. 5B,C). The cytotoxic mechanism underling acrolein-induced neuronal toxicity was investigated by measuring active caspase 3 (a biomarker of apoptosis) as well as receptor-interacting protein (RIP)1 and RIP3 (two biomarkers of necroptosis). Western blot assay demonstrated that acrolein concentration-dependently decreased pro-caspase 3 levels and increased active caspase 3 levels in primary cultured cortical neurons (Fig. 6A). Co-incubation of selumetinib inhibited acrolein-induced elevation in activated caspase 3 (Fig. 6B). Furthermore, acrolein significantly increased RIP1 and RIP3 in concentration-dependent manners (Fig. 6C). Selumetinib abolished acrolein-induced elevation in RIP1 and RIP3 (Fig. 6D). These data indicate that selumetinib prevented acrolein-induced programmed cell death, including apoptosis and necroptosis.

## Discussion

In the present study, the neuroprotective effect of selumetinib via a MEK–ERK inhibitory mechanism on acrolein-induced neurotoxicity was demonstrated in several ways. First, acrolein-induced elevation in phosphorylated ERK was consistently observed in primary cultured cortical neurons, indicating an involvement of MEK–ERK signaling in the acrolein-induced neurotoxicity. Furthermore, acrolein exerted its oxidative action by activating Nrf-2-HO-1 pathways as well as increasing FDP-lysine levels and α-synuclein aggregation. At the same time, acrolein damaged neurite outgrowth and induced both apoptosis and necroptosis. In the present study, we are the first to employ selumetinib as a neuroprotective agent against acrolein-induced neuronal toxicity by demonstrating that selumetinib attenuated acrolein-induced oxidative injury, protein conjugation and aggregation, neurite damages as well as programmed cell death. These data suggest that selumetinib, a MEK–ERK inhibitor, may be translationally employed for inhibiting acrolein-induced neurotoxicity in CNS neurodegenerative diseases.

Acrolein levels in the brain of Alzheimer’s patients is approximately 70–500 μM which is about 140–1000 folds higher than that in healthy subjects^25^. Previously, the LD_50_ of acrolein was reportedly 600 μM in mouse sensory neurons^26^. In contrast, our LDH assay showed a much lower LD_50_ of acrolein, i.e., 30 μM, in primary cultured cortical neurons. Furthermore, we found that 10–30 μM acrolein consistently induced ERK phosphorylation and neurotoxicity in primary cultured cortical neurons. Therefore, we employed a sub-lethal level of acrolein (30 μM) to investigate the neuroprotective effect of selumetinib on acrolein-induced neurotoxicity. Morphologically, acrolein concentration-dependently damaged neurite outgrowth, including neuritic beading, neurite discontinuation and reduction in neurite extension. Our morphological data are consistent with a previous study demonstrating that acrolein caused fewer neurite retention on the swelling neurons^26^. In addition, we observed severe damages of neuronal cell body when subjected to 20–30 μM acrolein. The discrepancy of acrolein-induced neurotoxicity between LDH cytotoxicity assay and neurite outgrowth assay may be due to the cell density (5 × 10^6^ cells in the LDH assay vs. 3 × 10^5^ cells in the immunostaining study) since the lower density of seeding cells, the higher cytotoxicity is induced^27,28^. Moreover, our data showed that acrolein is capable of inducing HO-1, FDP-lysine and α-synuclein aggregation as well as apoptosis and necroptosis. These in vitro data further support our previous in vivo study demonstrating that intranigral infusion of acrolein elevated oxidative stress, protein conjugation and aggregation as well as dopaminergic cell loss in the affected nigrostriatal dopaminergic system of rat brain^11^, suggesting a universal acrolein-induced neurotoxicity.

Due to the neuropathological role of acrolein, a significant body of studies has proposed neuroprotective strategies to inhibit acrolein-induced neurotoxicity. Because of its highly reactive property, potential therapeutic agents with an anti-oxidative activity, including curcumin and caffeic acid, have been reported to reduce acrolein-induced oxidative injuries and cell death in vitro^6,19,23^. Furthermore, oral administration of baicalein and crocin via anti-oxidative mechanisms attenuated acrolein-induced neurotoxicity in Parkinsonian and Alzheimer’s animal models, respectively^29,30^. In the present study, we targeted the MEK–ERK signaling pathway in acrolein-induced neurotoxicity but not PIK3-AKT signaling pathway because acrolein did not consistently alter AKT signaling^6,23^. Furthermore, LY294002, a PI3K inhibitor reportedly enhanced acrolein-induced apoptosis in SH-SY5Y cells^6^. Selumetinib as a potential therapeutic was selected for a number of reasons. First, in contrast to PD98059 and U0126 which are laboratory grade chemicals^5,6^, selumetinib is a leading anti-cancer drug currently being used in clinical trials. Secondly, using iron-induced lipid peroxidation in brain homogenates, we did not detect any anti-oxidative activity of selumetinib (data not shown), indicating that selumetinib indeed exerts its neuroprotective action via blocking acrolein-activated MEK–ERK signaling rather than scavenging ROS. Thirdly, in addition to the present study, our previous study has reported that selumetinib significantly inhibited acrolein-induced inflammasome formation and activation of BV-2 cells, an alternative model for primary cultured microglia^9^. Due to its moderate permeability of the blood brain barrier^31^ and demonstrated safety when administered orally for 14 days^30^, a systemic application of selumetinib may be beneficial to patients with acute stroke stage and SCI via inhibiting both acrolein-induced toxicity to neurons and acrolein-induced neuroinflammation in microglia as well. Currently, an in vivo study focusing on the neuroprotective effect of oral administration of Zorifertinib (an epidermal growth factor receptor-tyrosine kinase inhibitor) is ongoing to demonstrate the application of cancer target therapy for acute brain damages.

In conclusion, the present study and our previous studies have demonstrated that acrolein induces neurotoxicity and neuroinflammation via activating MEK–ERK signaling in neurons and BV-2 cells, respectively. Furthermore, our data show that selumetinib consistently inhibits acrolein-induced neurotoxicity and neuroinflammation, suggesting a novel drug repurposing use of selumetinib for CNS neurodegenerative diseases.

## Materials and methods

### Drugs

The chemicals used were acrolein (Sigma, St. Louis, MO, USA) and selumetinib (Abmole Bioscience, Houston, USA). Selumetinib was dissolved in dimethyl sulfoxide (DMSO, Sigma, St. Louis, MO, USA) and was diluted with DMEM or Neurobasal (NB, Thermo Fisher Scientific, Waltham, MA, USA) medium.

### Cultured cells

Rat primary cultured cortical neurons were prepared from embryonic day 17 rat brains^32^. The dissociated cells suspended in the Basal Medium Eagle (BME, Thermo Fisher Scientific, Waltham, MA, USA) medium containing 20% fetal bovine serum, were seeded onto 35-mm culture dish (IWAKI, Tokyo, Japan) with a density of 5 × 10^6^ cells per dish. Afterwards, cells were maintained with serum-free NB medium supplemented with B27 (Thermo Fisher Scientific, Waltham, MA, USA) in the incubator with 5% CO_2_ at 37 °C. The use of animals has been approved by the Institutional Animal Care and Use Committee of Taipei Veterans General Hospital, Taipei, Taiwan. The methods were carried out in accordance with the approved guidelines. All experiments were performed in the accordance with relevant guidelines and regulation. The approval number is IACUC2017-242. This was an in vitro study and did not fit ARRIVE guidelines.

### Cytotoxicity assay

Cytotoxicity was determined by measuring LDH in the culture supernatant^33^. In brief, primary cultured cortical neurons (5 × 10^6^ cells /well) were seeded on a 24-well plate and treated with acrolein for 24 h. The LDH secreted in the culture medium was assessed by addition of β-NADH and sodium pyruvate (Sigma, St. Louis, MO, USA). LDH activity was determined by measuring the absorbance at 340 nm for 6 min using an ELISA reader (TECAN Sunrise, Männedorf, Schweiz). The LDH activity of cells treated with 0.1% Triton X-100 was used as control set to 100%.

### The cell viability assay

Cell viability was determined using a modified 3-(4,5-dimethylthiazol-2-yl)-2,5-diphenyl tetrazolium (MTT; Sigma, St. Louis, MO, USA) assay^34^. In brief, the culture medium was removed and the cells were washed with phosphate-buffered saline (PBS). The cells were then incubated with MTT solution (5 mg/mL in PBS) for 3 h. Afterwards, the MTT solution was removed and the resulting formazan was dissolved with DMSO (100 μL). The absorption was measured at 570 nm with a reference wavelength of 630 nm.

### Western blots analysis

Western blot assay was performed as follows^33^. At the end of acrolein treatment, the cells were collected, washed with phosphate buffered saline (PBS), and lysed in radioimmunoprecipitation assay (RIPA, Cell Signaling Tech. Beverly, MA, USA) lysis buffer containing 20 mM Tris HCl, 150 mM NaCl, 1% (v/v) NP-40, 1% (w/v) sodium deoxycholate, 1 mM ethylenediaminetetraacetates (EDTA), 0.1% (w/v) sodium dodecyl sulfate polyacrylamide (SDS) and 0.01% (w/v) sodium azide (pH 7.5) for 20 min on ice. Lysates were then centrifuged at 12,000 rpm for 10 min, and the protein concentrations of supernatant were determined by Pierce BCA Protein Assay Kit (Thermo Fisher Scientific, Waltham, MA, USA). Protein samples (30 μg) were run on 8–13.5% SDS-polyacrylamide gel electrophoresis and then transferred onto a polyvinylidene difluoride (PVDF, Bio-Rad, USA.) at 100 V for 120 min. Blots were probed with primary antibodies including antibodies against HO-1 (StressGen, Victoria, CA, USA), FDP-lysine, Nrf-2 (Abcam, Cambridge, UK), caspase 3, phospho-ERK, total-ERK, α-synuclein, RIP1, RIP3 (Cell Signaling Tech. Beverly, MA, USA) overnight at 4 °C. The secondary antibodies were horseradish peroxidase-conjugated secondary IgG (Chemicon, Temecula, CA, USA). After primary antibody incubation, the membrane was washed and incubated with a secondary antibody for 1 h at room temperature. The immunoreaction was visualized using Amersham Enhanced Chemiluminescence (Amersham Pharmacia Biotech, Piscataway, NJ, USA). After this detection, the bound primary and secondary antibodies were stripped by incubating the membrane in stripping buffer (100 mM 2-mercaptoethanol, 2% SDS) at 50 °C for 5 min. The membrane was reprobed with a primary antibody against β-actin (Millipore, Billerica, MA, USA).

### Neurite outgrowth assay

Primary cultured cortical neurons were cultured on Transwell inserts with 1 μm pore diameter inside 24-well plates (IWAKI, Tokyo, Japan) in NB medium^35^. Neurites passed through the porous membrane and grew parallel to cell body layer for 4 days. After wiping off the cell bodies inside the transwell, the extending neurites on the other side of lower chamber were immunostained with microtubule-associated protein 2 (MAP-2, Cell Signaling Tech. Beverly, MA, USA). The immunoreactivity was quantified using image J.

### Immunohistochemical staining

Primary cultured cortical neurons (3 × 10^5^ cells) were grown in 35 mm petri dish containing coverslips. At the end of acrolein treatment, the cells on the coverslips were fixed with 4% paraformaldehyde (Merck, Whitehouse Station, NJ, USA), cultured cells and then were washed with 0.1 M PBS, incubated with 0.3% Triton X-100 (Sigma, St. Louis, MO, USA) and 1% goat serum (GS; Jackson ImmunoResearch. PA, USA), and blocked with 3% GS for 60 min. Next, cells were processed for immunostaining using mouse monoclonal antibody specific for rat anti-GAP-43, Neu-N (Millipore, Beverly, MA, USA) in 1% GS-PBS at 4 °C for 24 h. The cells were then incubated in fluorescein conjugated-IgG (FITC) (Jackson ImmunoResearch. PA, USA) and Texas Red dye-conjugated IgG fraction monoclonal mouse anti-biotin (Jackson ImmunoResearch. PA, USA) for 1 h at room temperature, mounted in glycerol (Merck, MA, USA) and visualized by a fluorescence confocal microscope (Olympus FluoView, Norfolk, VA, USA).

### Statistics

All data are expressed as the mean ± SEM. The results of Western blot assays of were analyzed by one-way analysis of variance (one-way ANOVA) and followed by the LSD test as post-hoc method.

### Ethics approval and consent to participate

The use of animals has been approved by the Institutional Animal Care and Use Committee of Taipei Veterans General Hospital, Taipei, Taiwan, R.O.C.

## Supplementary Information

Supplementary Figures.
