# Supplementary material for: Neuroprotective effect of selumetinib on acrolein-induced neurotoxicity
Source: Sci Rep. 2021 Jun 14;11:12497. doi: 10.1038/s41598-021-91507-6 (PMC8203693; doi:10.1038/s41598-021-91507-6)

Fig1a Acrolein-elevated ERK phosphorylation: time-dependence

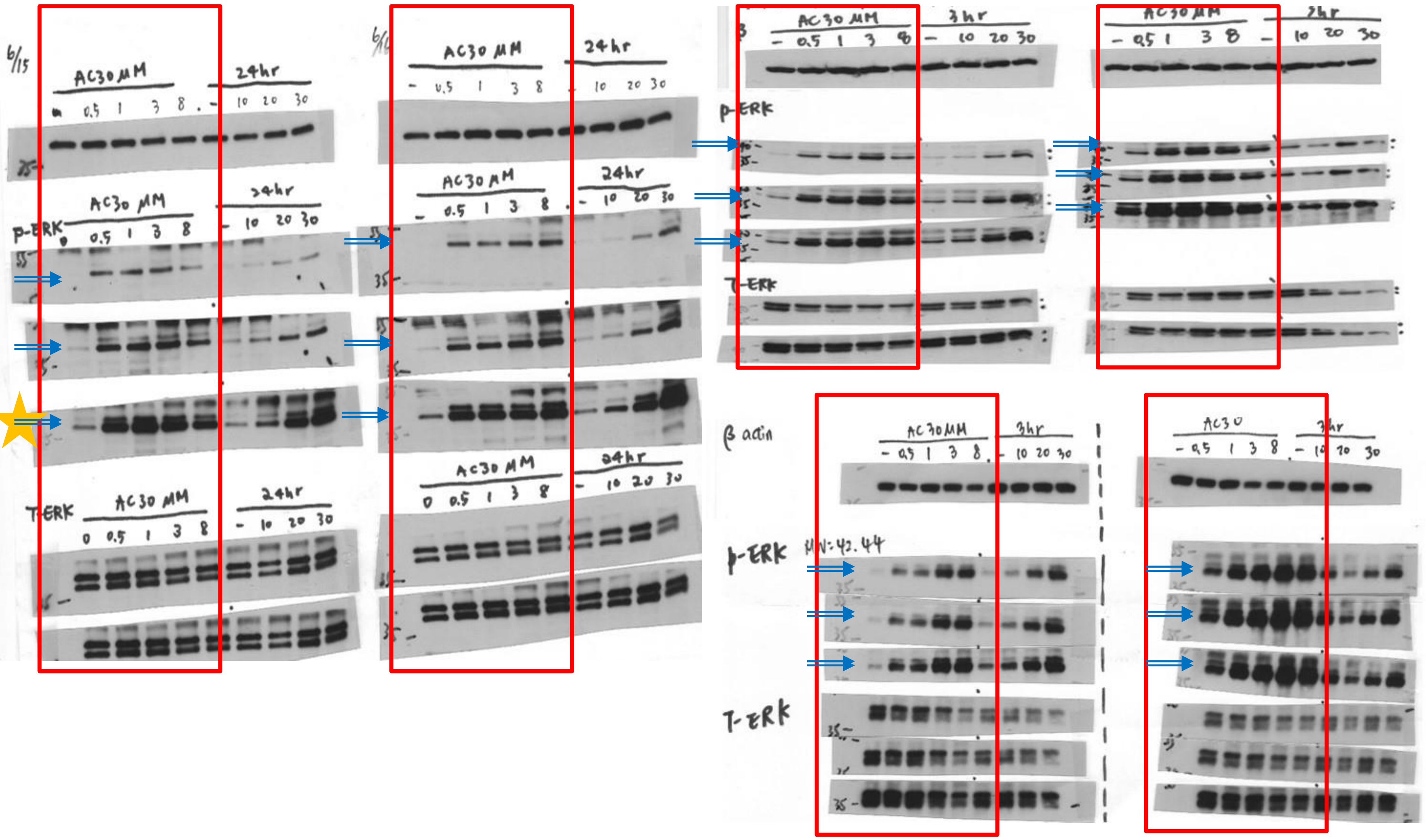

Fig1b Acrolein-elevated ERK phosphorylation: concentration-dependence

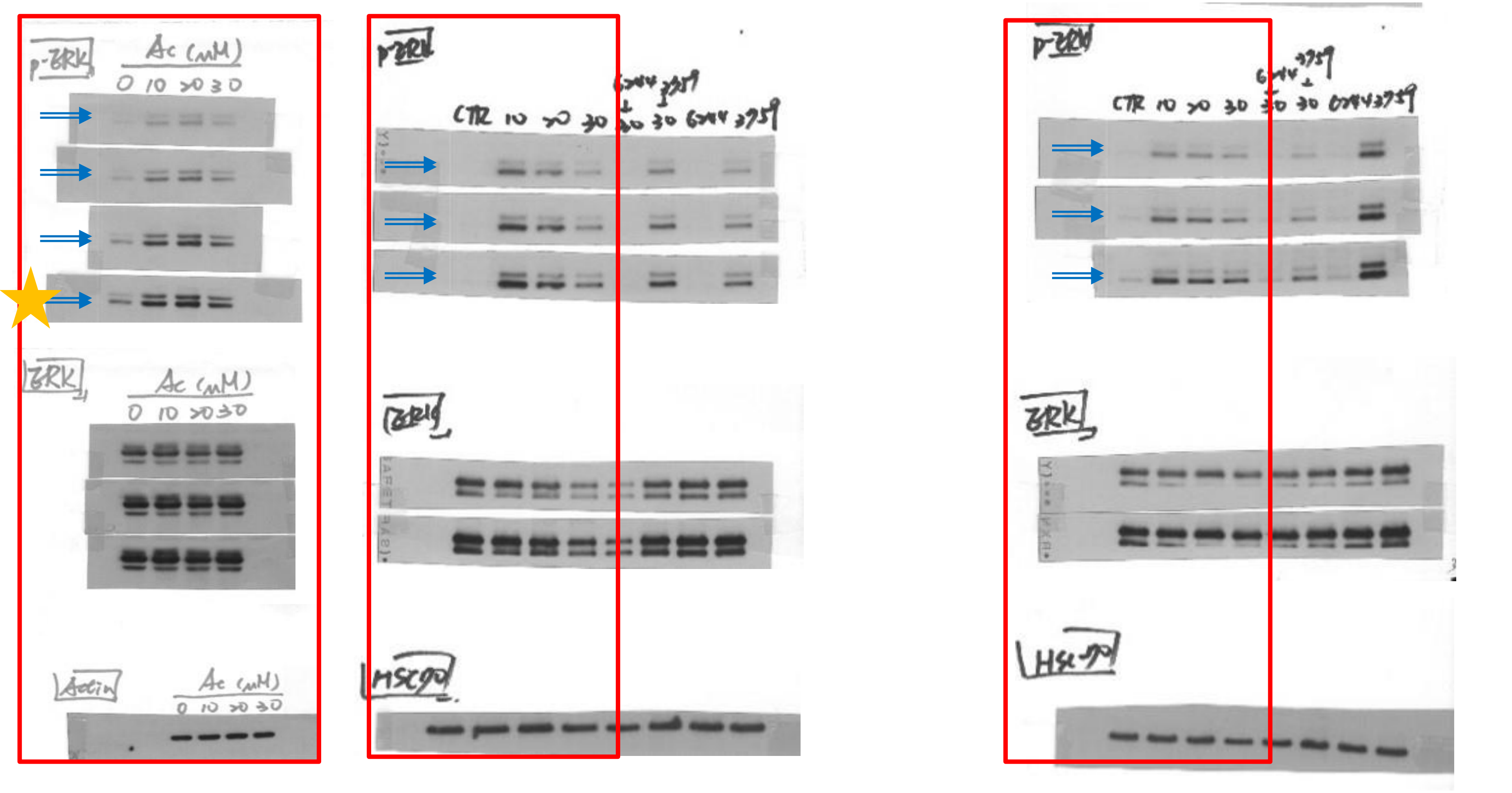

Fig1c Effect of selumetinib on acrolein-elevated ERK phosphorylation

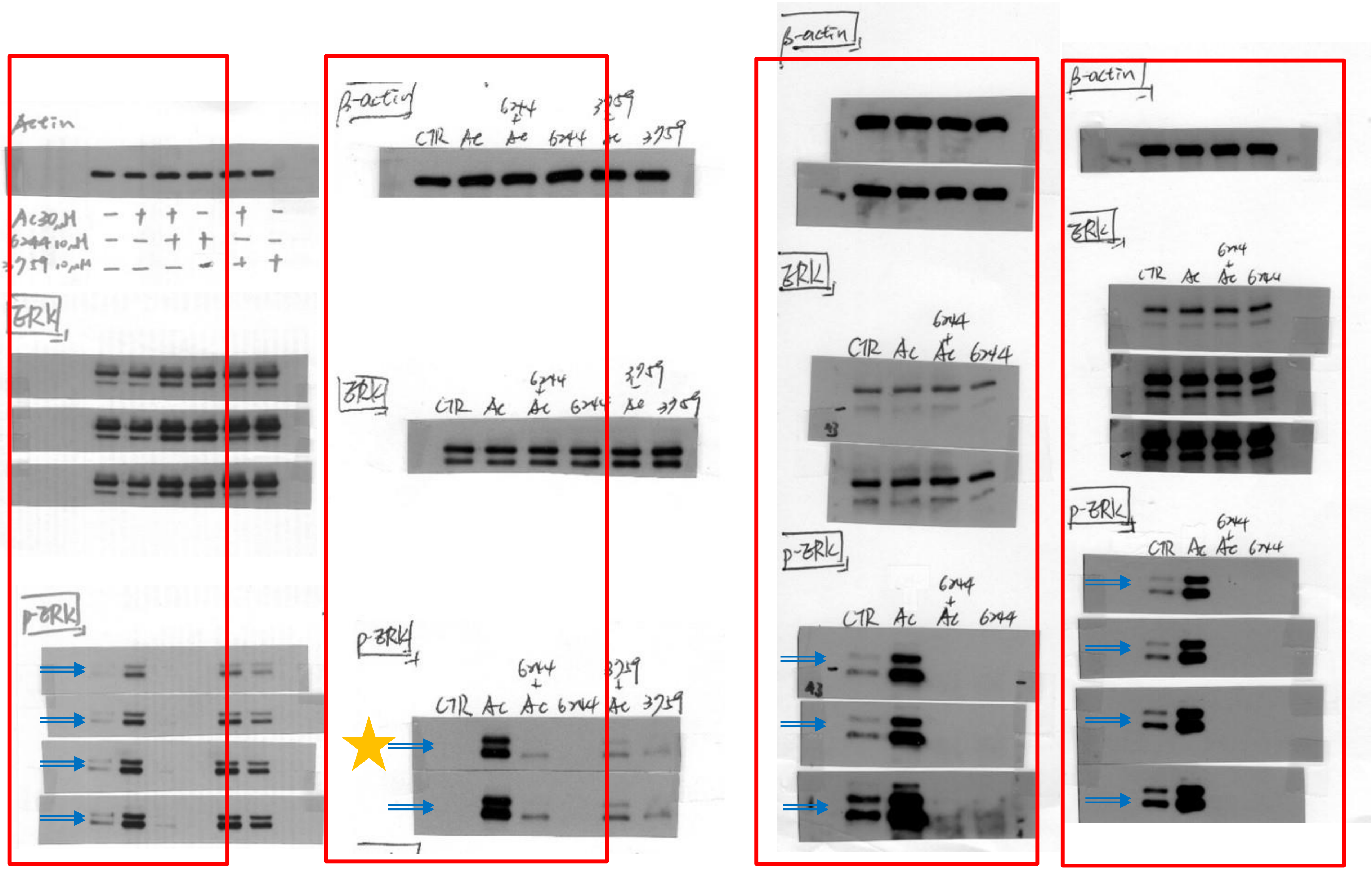

Fig2a Acrolein-induced HO-1 elevation: concentration-dependence

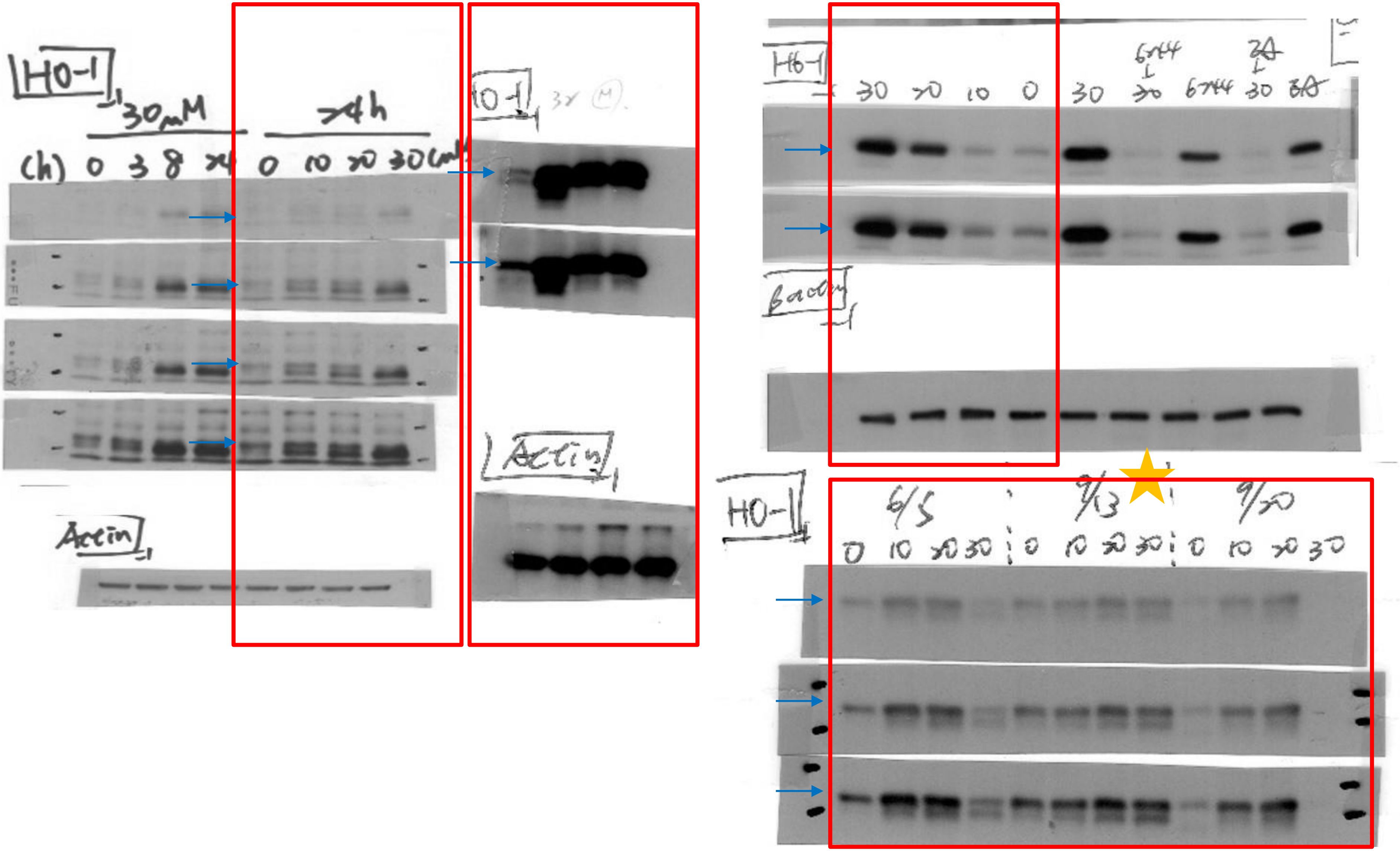

Fig2b Effect of selumetinib on acrolein-elevated Nrf-2

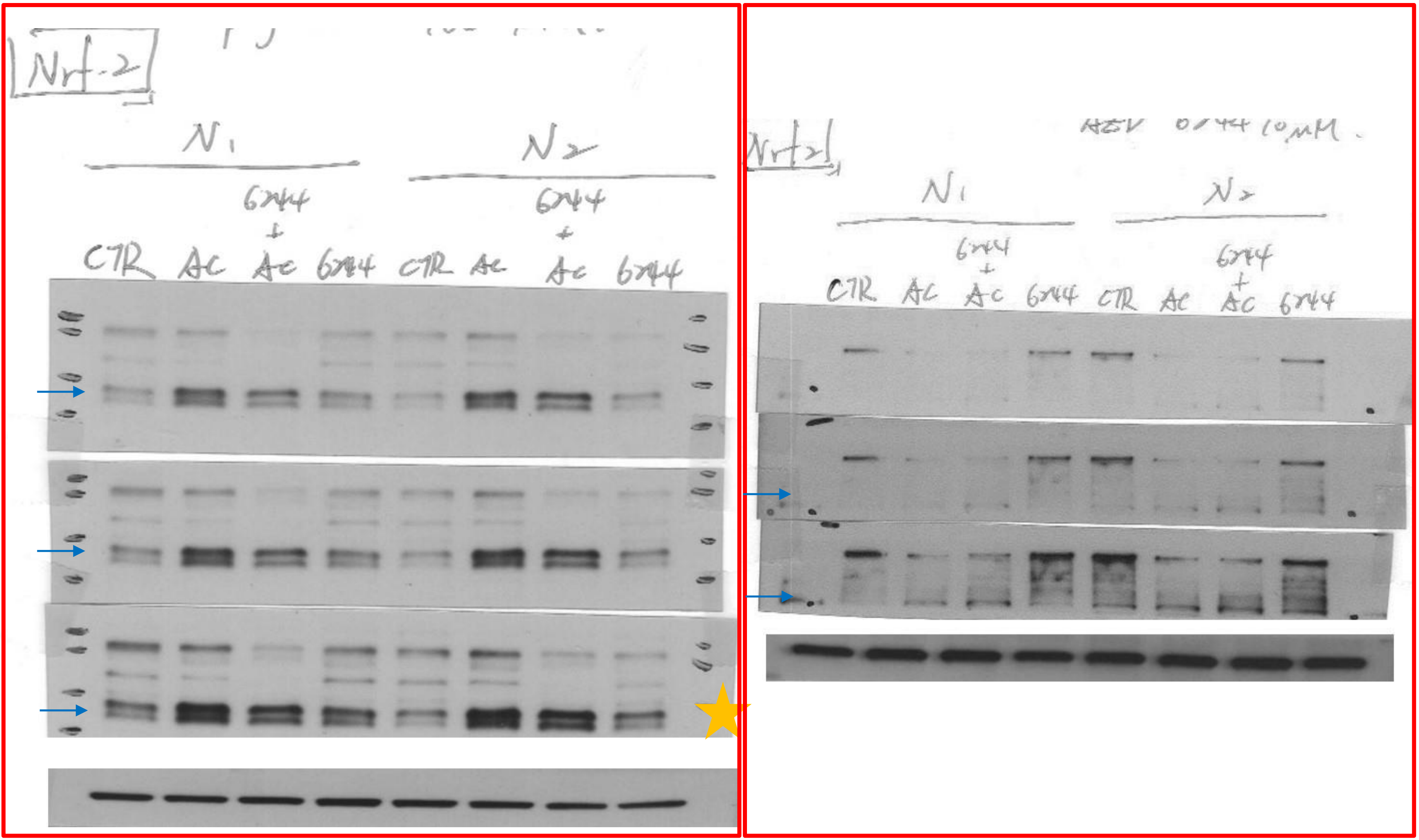

Fig2c Effect of selumetinib on acrolein-elevated HO-1

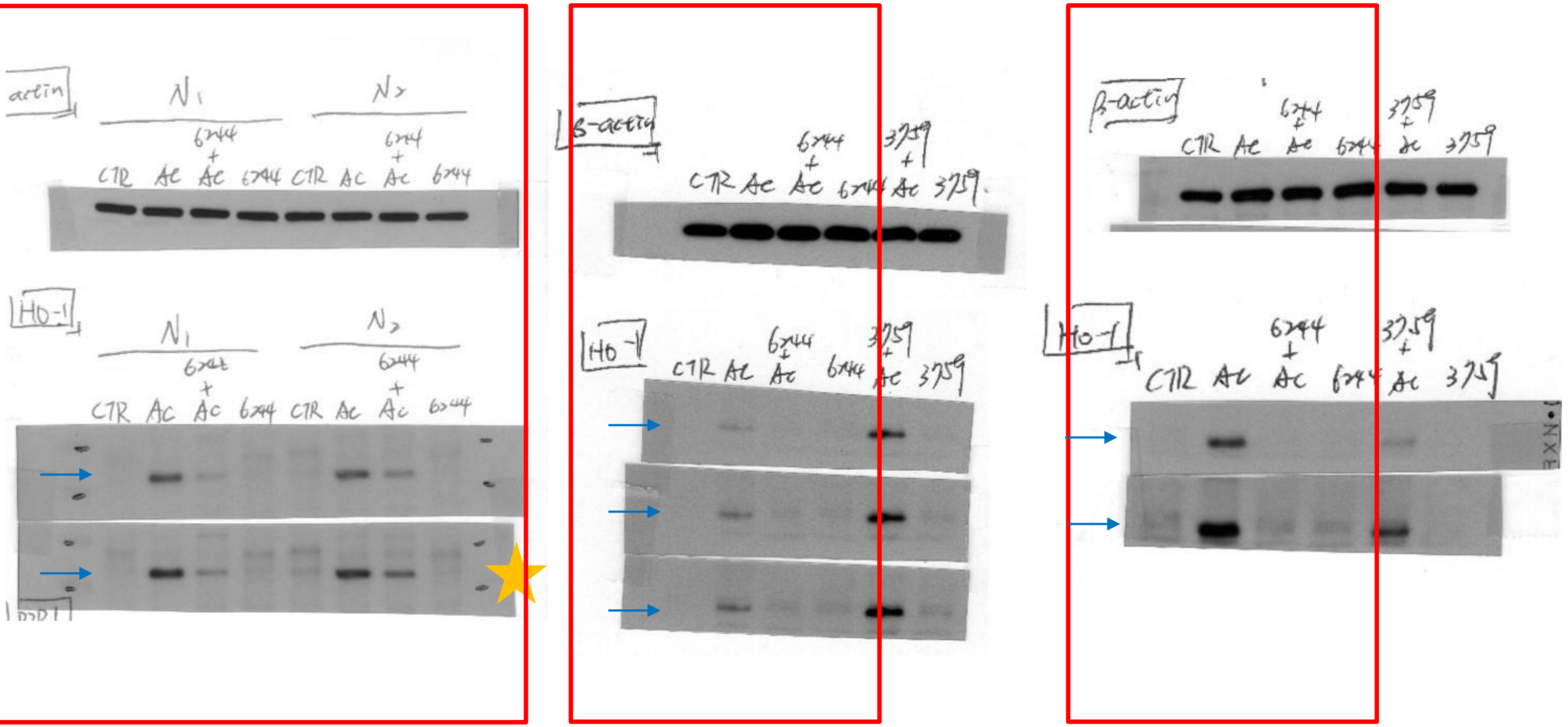

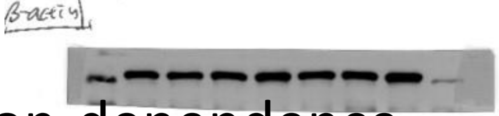

Fig3a Acrolein-induced Ac-FDP: concentration-dependence

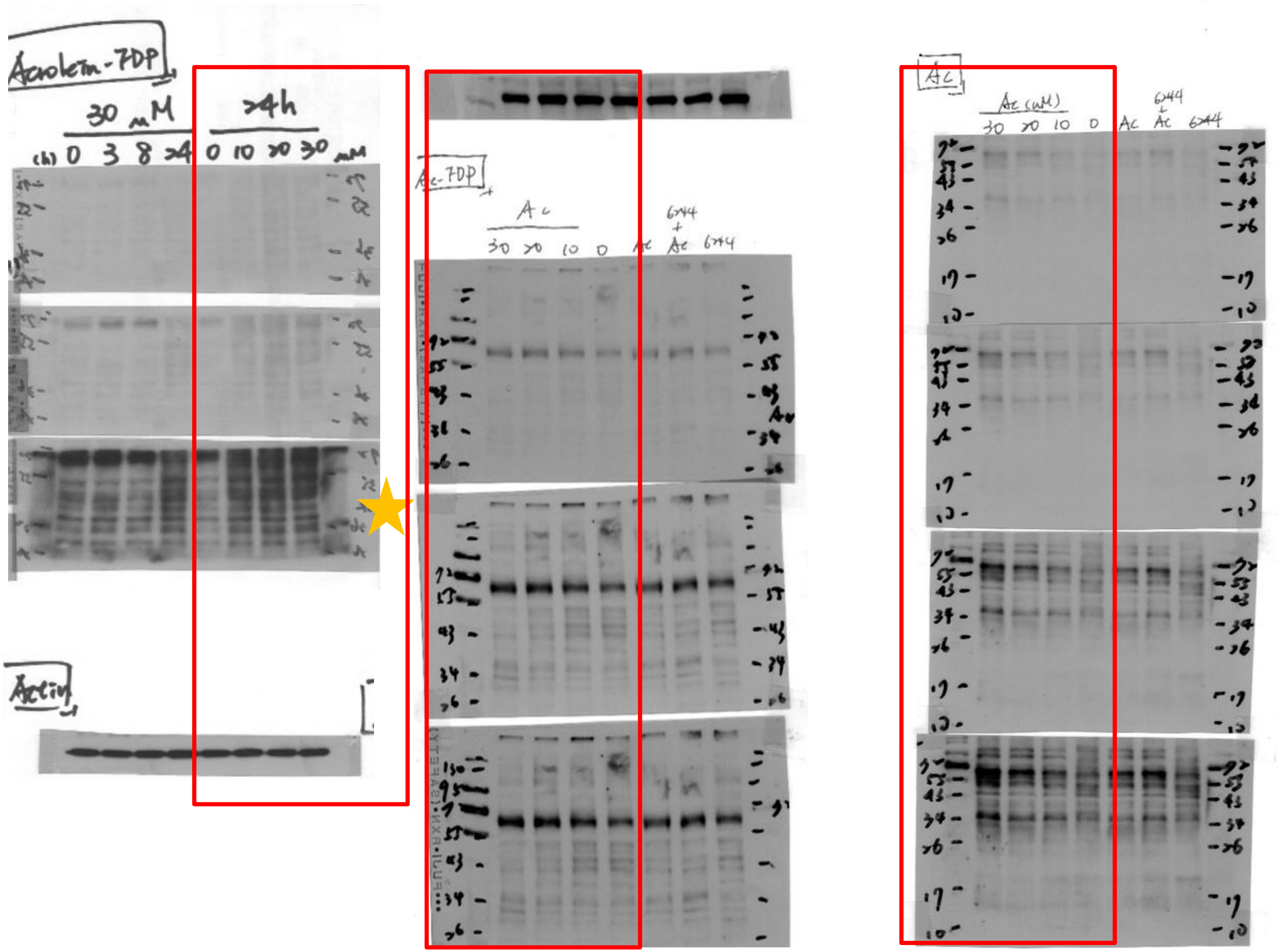

Fig3b Effect of selumetinib on acrolein-elevated Ac-FDP

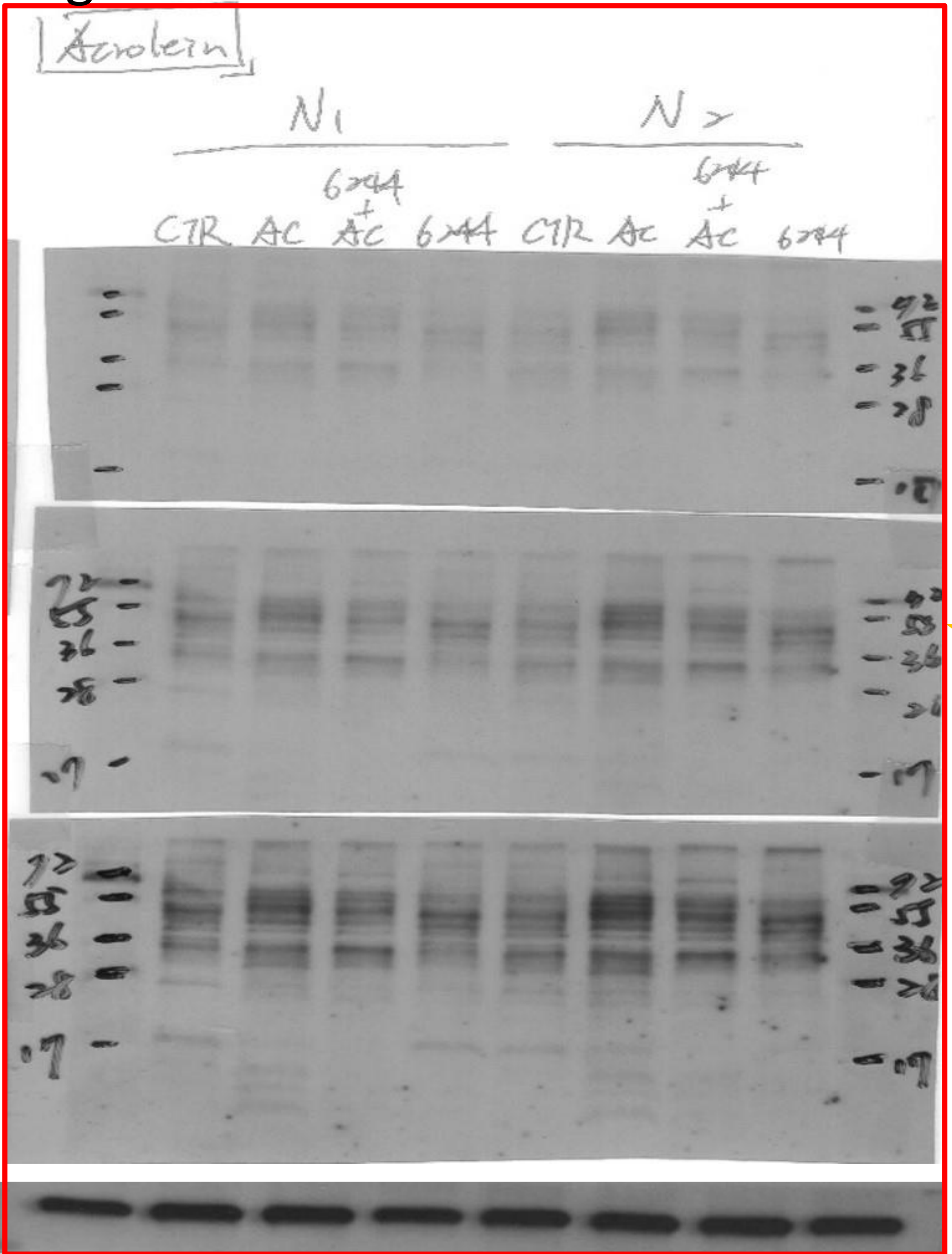

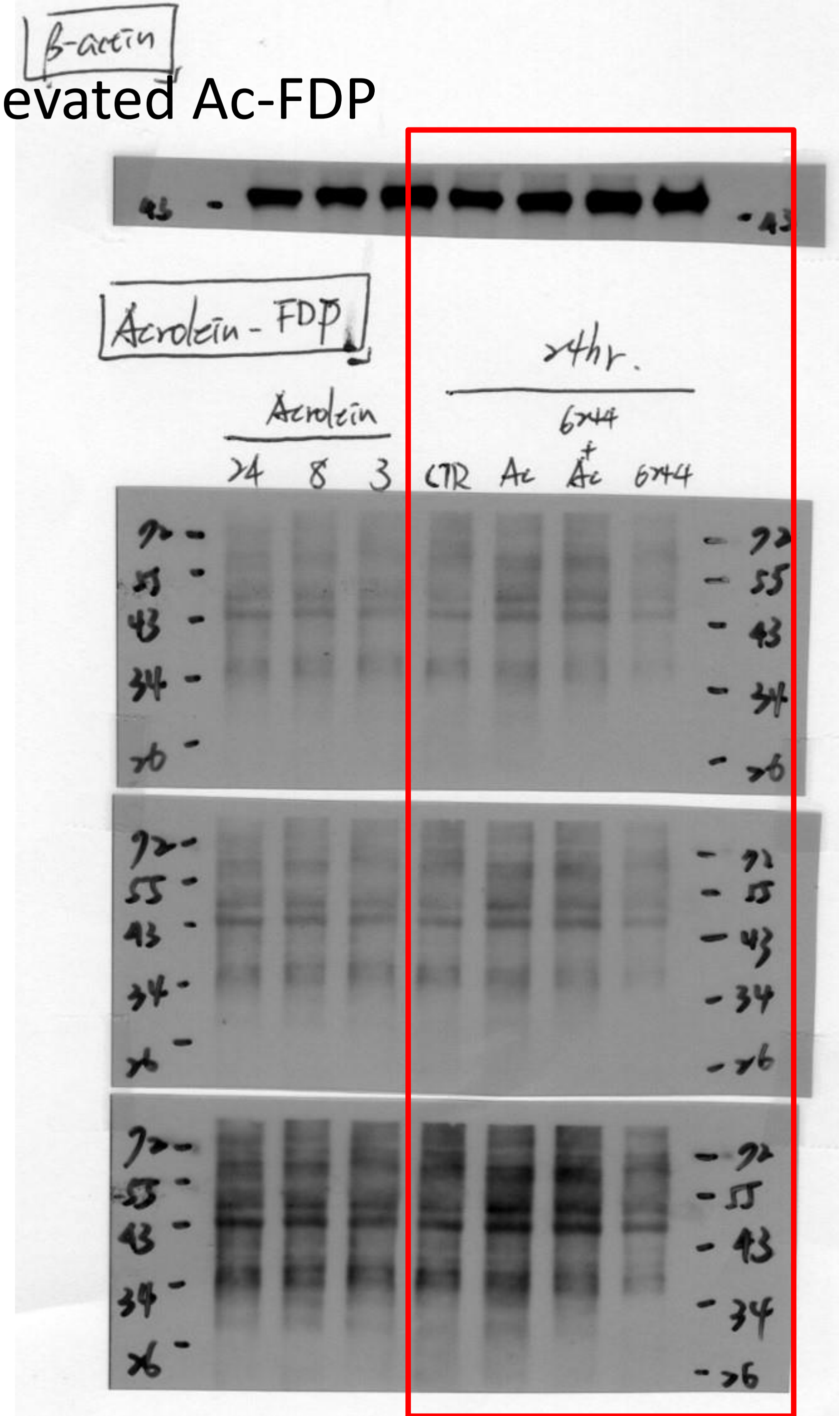

Fig3c Effect of selumetinib on acrolein-elevated α-synuclein aggregates

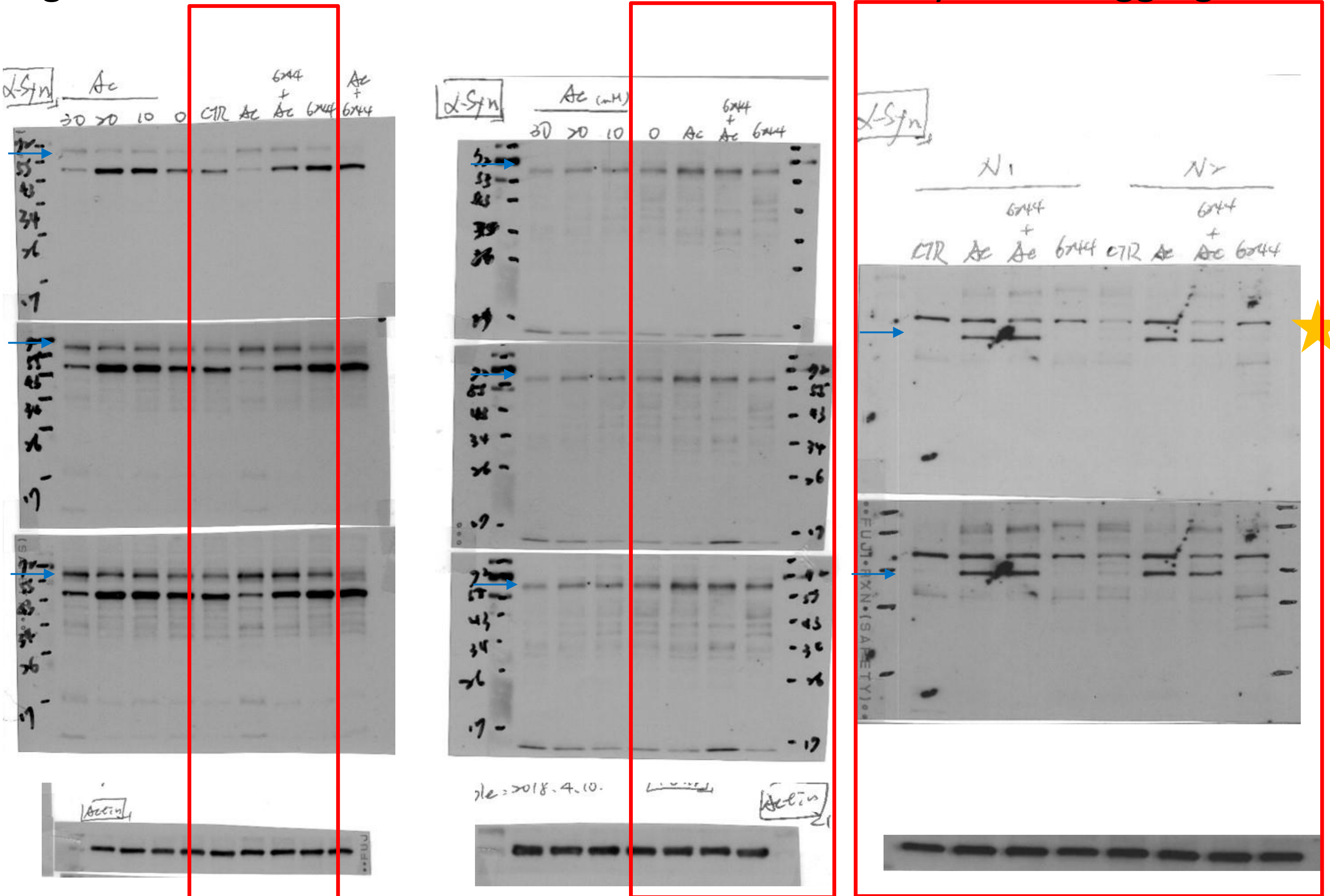

Fig6a Acrolein-induced apoptosis: concentration-dependence

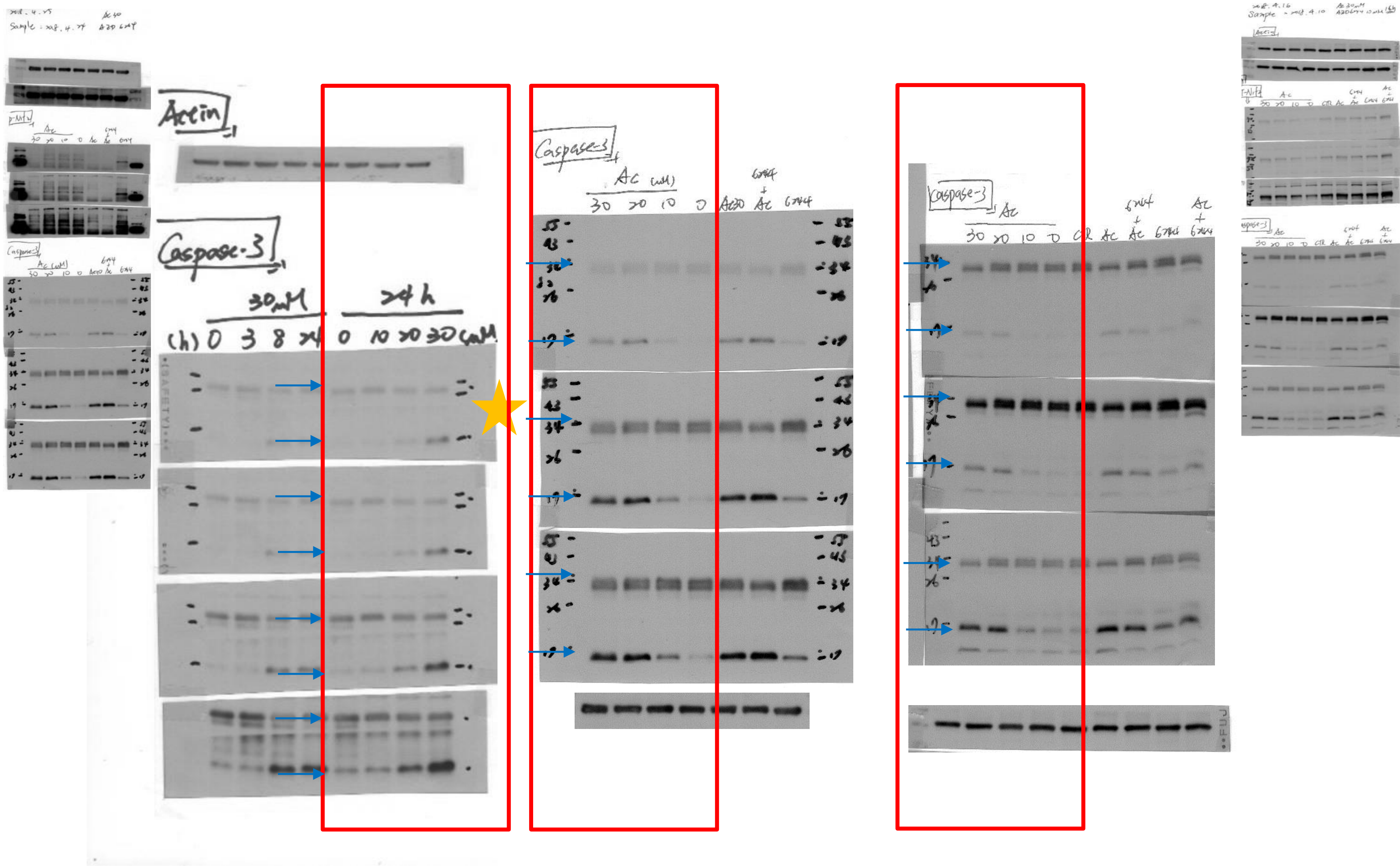

Fig6b Effect of selumetinib on acrolein-induced apoptosis

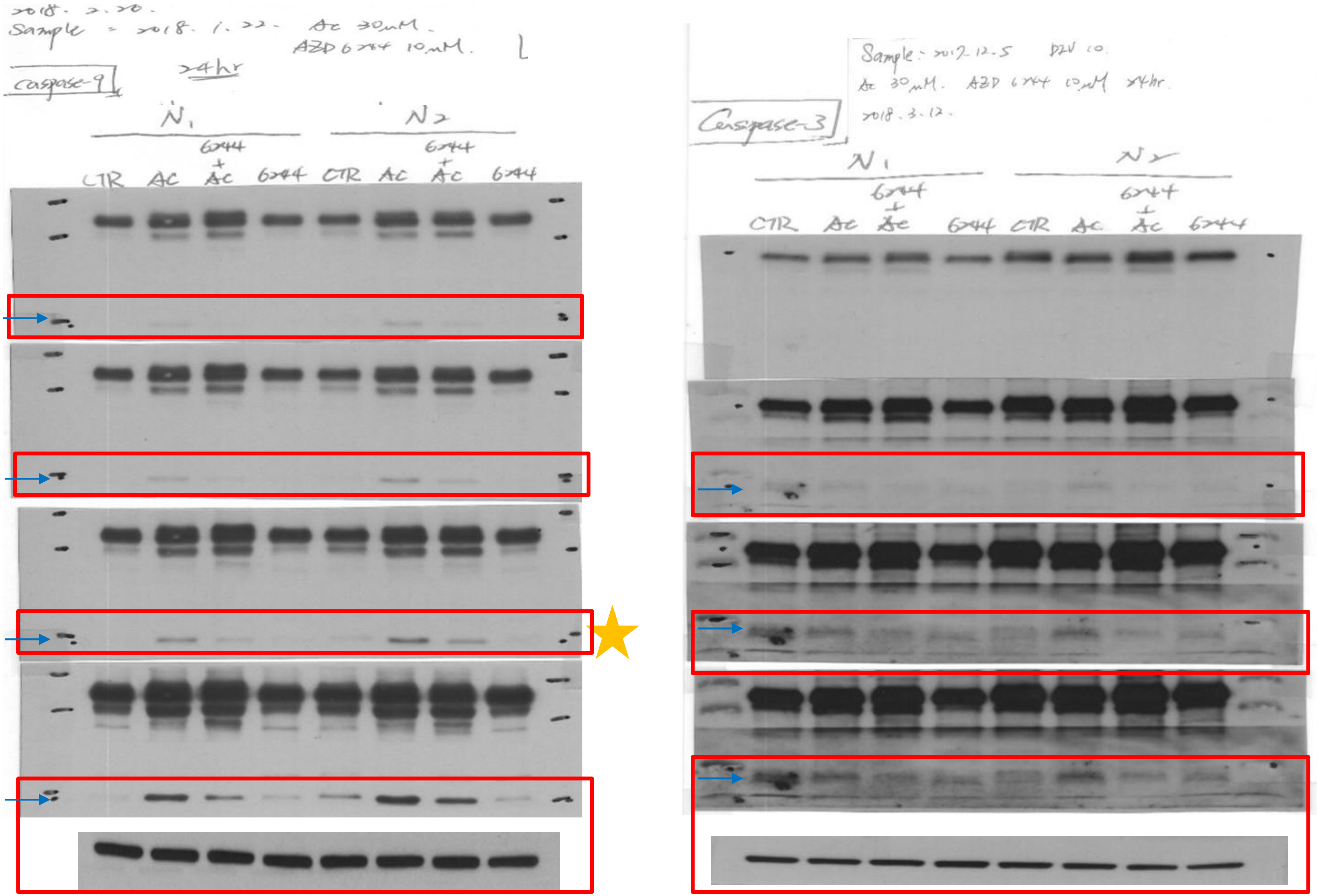

Fig6c Acrolein-induced necroptosis: concentration-dependence

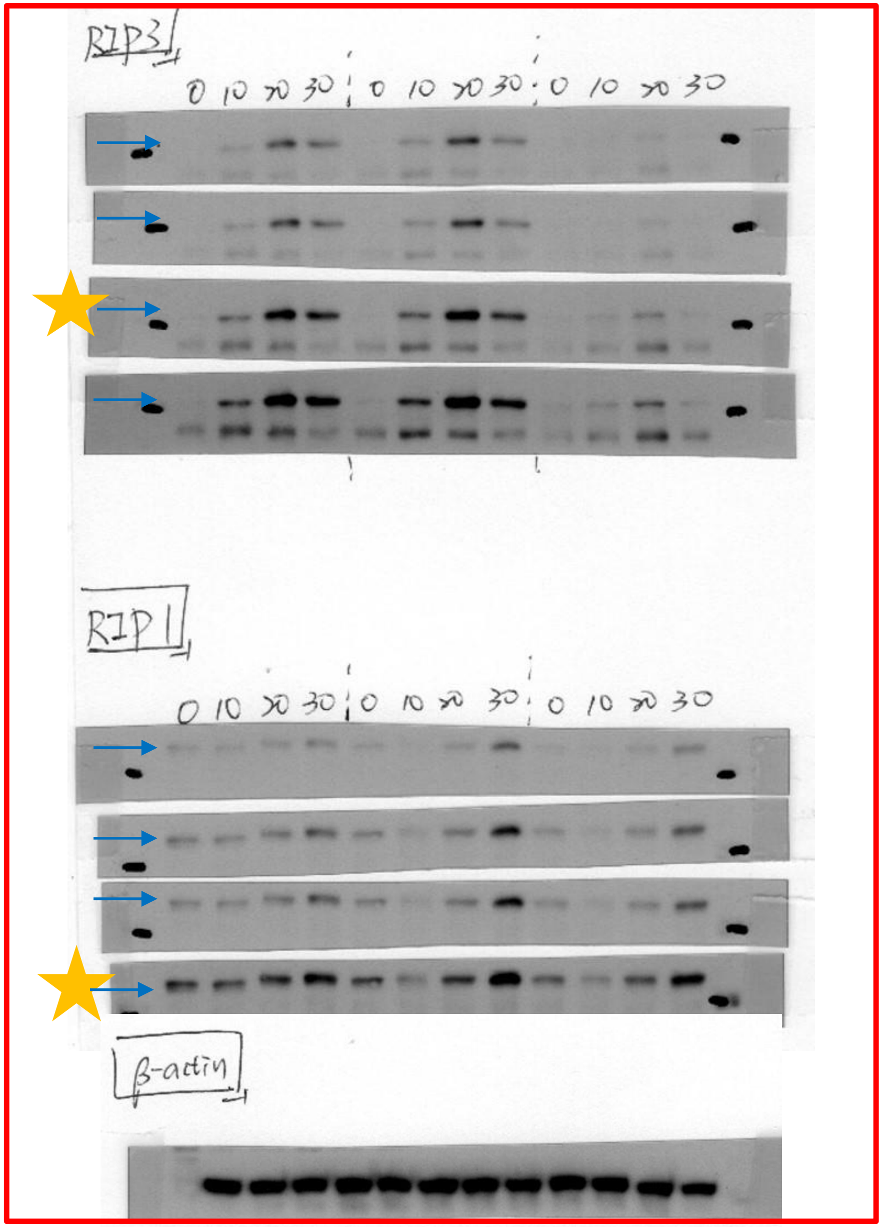

Fig6d Effect of selumetinib on acrolein-induced necroptosis

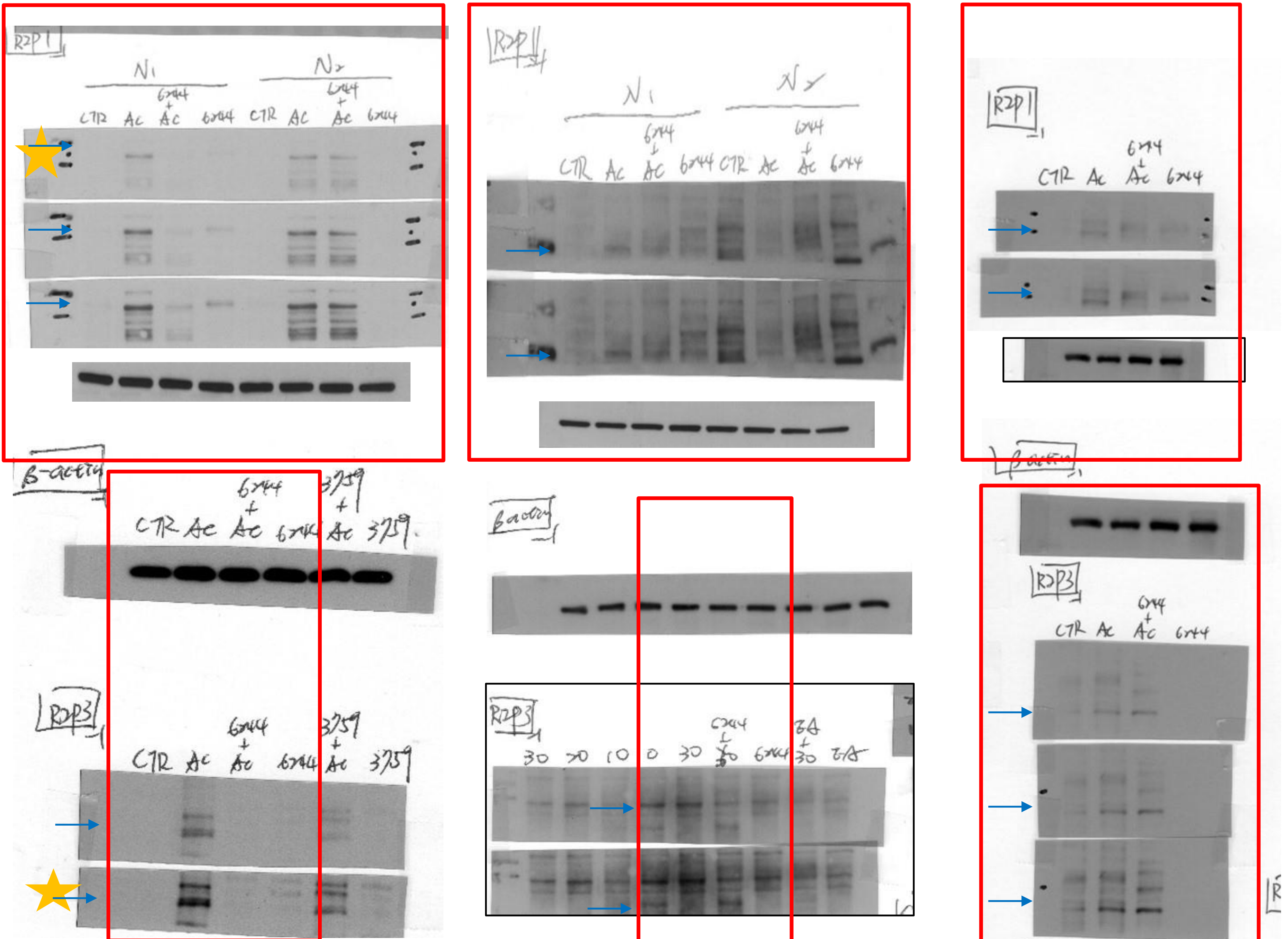

Supplement: Supplementary file 1 — Supplementary Figures. [file 41598_2021_91507_MOESM1_ESM.pdf]
